# Near Room Temperature Light-Activated WS_2_-Decorated rGO as NO_2_ Gas Sensor

**DOI:** 10.3390/s19112617

**Published:** 2019-06-09

**Authors:** Valentina Paolucci, Seyed Mahmoud Emamjomeh, Luca Ottaviano, Carlo Cantalini

**Affiliations:** 1 Department of Industrial and Information Engineering and Economics, Via G. Gronchi 18, University of L’Aquila, I-67100 L’Aquila, Italy; seyedmahmoud.emamjomeh@graduate.univaq.it (S.M.E.); carlo.cantalini@univaq.it (C.C.); 2 Department of Physical and Chemical Sciences, Via Vetoio 10, University of L’Aquila, I-67100 L’Aquila, Italy; luca.ottaviano@aquila.infn.it; 3 CNR-SPIN Uos L’Aquila, Via Vetoio 10, I-67100 L’Aquila, Italy

**Keywords:** WS_2_–rGO hybrids, chemoresistive sensors, NO_2_, Purple Blue light activation

## Abstract

The NO_2_ response in the range of 200 ppb to 1 ppm of a chemoresistive WS_2_-decorated rGO sensor has been investigated at operating temperatures of 25 °C and 50 °C in dry and humid air (40% RH) under dark and Purple Blue (PB) light conditions (λ = 430 nm). Few-layers WS_2_, exfoliated by ball milling and sonication technique, with average dimensions of 200 nm, have been mixed with rGO flakes (average dimension 700 nm) to yield WS_2_-decorated rGO, deposited on Si_3_N_4_ substrates, provided with platinum (30 μm gap distance) finger-type electrodes. TEM analysis showed the formation of homogeneous and well-dispersed WS_2_ flakes distributed over a thin, continuous and uniform underlying layer of interconnected rGO flakes. XPS and STEM revealed a partial oxidation of WS_2_ flakes leading to the formation of 18% amorphous WO_3_ over the WS_2_ flakes. PB-light irradiation and mild heating of the sensor at 50 °C substantially enhanced the baseline recovery yielding improved adsorption/desorption rates, with detection limit of 400 ppb NO_2_ and reproducible gas responses. Cross sensitivity tests with humid air interfering vapor highlighted a negligible influence of water vapor on the NO_2_ response. A charge carrier mechanism between WS_2_ and rGO is proposed and discussed to explain the overall NO_2_ and H_2_O response of the WS_2_–rGO hybrids.

## 1. Introduction

The intrinsic merits of Transition Metal Dichalcogenides (TMDs), including their high surface-to-volume ratio and semiconducting properties, have accelerated the development of a diverse range of applications of these materials as chemical sensors [1,2]. Two-dimensional (2D) mono- or few-layer TMDs produced by different exfoliation procedures, expose both plate and edge atoms of a single layer capable of adsorbing gas molecules, providing the largest sensing area per unit volume. Among a large variety of gaseous species, NO_2_, H_2_ and NH_3_ are the most investigated gases, considering their high chemical reactivity with mono- or few-layer MoS_2_ [3,4,5], WS_2_ [6,7], MoSe_2_ [8] and MoTe_2_ [9].

In need to decrease the operating temperature of the sensing materials, a common drawback of TMDs operating at room temperature, is their slow response and recovery times, or even no recovery when used at low temperatures [3,5,10]. The increase of the operating temperature up to 150 °C greatly improves adsorption/desorption rates and baseline recovery, but causes partial oxidation of TMDs into their metal oxide counterparts, as previously demonstrated for MoS_2_ and WS_2_-layered materials [5,7,11]. It turns out that both low and high temperatures hinder the practical use of TMDs due to kinetic (i.e., slow recovery rates) and thermodynamical (i.e., spontaneous oxidation) reasons, respectively. A possible strategy to avoid irreversible adsorption and ageing phenomena, and thus, to enhance the long term stability of the sensors, is to operate them at low temperature utilizing light irradiation as an external source of energy, as previously reported for metal oxide sensors [12,13], rGO-Metal Oxide nanocomposite [14], graphene [15], MoS_2_ [3] and WS_2_ [16].

Considering the variety of different preparation techniques for producing mono- or few-flake TMDs, consistent with standard electronic processes, some other aspects must be considered. A first issue is the reproducibility of the exfoliation procedure with respect to both microstructure (i.e., number of layers, lateral size, surface area, etc.) and chemical composition (i.e., defects concentration and surface oxidation). A second aspect is the small allowable average lateral size of the TMDs flakes, which may range from 100 to 300 nm, depending on the exfoliation technique [17]. A final issue is the surface coverage of the substrate, needed to create percolation paths for charge carriers between TMDs flakes, bridging metal electrodes. The possibility of using hybrid nanostructures, mostly focused on MoS_2_/graphene, making use of large-size, conducting-flakes-pathways of Graphene Oxide (GO) or reduced Graphene Oxide (rGO) with dispersed smaller-size, lesser-conductive-flakes of MoS_2_ has been recently reported as an effective solution to enhance the fabrication of this new class of gas sensors [18,19,20]. Beside some reports on the utilization of WS_2_/GO hybrids as electrocatalysts for hydrogen evolution reactions [21,22] and WS_2_/GO as humidity [23] and NH_3_ [24] sensors, no applications of WS_2_–rGO nanocomposite for NO_2_ gas sensing applications have been reported so far.

In this paper, we report the exfoliation of WS_2_ powders by a combined ball milling and sonication technique, which leads to average dimensions of 200 nm and “aspect ratios”, i.e., lateral dimension to the thickness of 27, to produce mono to few-layer WS_2_ with controlled morphology and chemical composition. WS_2_ flakes are mixed with rGO flakes with average dimensions of 700 nm to yield WS_2_-decorated rGO as chemoresistive NO_2_ thin films deposited on large-area Si_3_N_4_ substrates, provided with platinum finger-type patterned electrodes.

The aim of this paper is firstly to demonstrate the reliability of the decoration process leading to the deposition of thin films of well dispersed WS_2_ flakes over large-size, interconnected rGO flakes, secondly, to demonstrate and discuss the influence of purple blue light (λ = 430 nm) to detect NO_2_ gas in air in the operating temperature range of 25 °C to 50 °C, and lastly, to investigate the influence of water vapor on the NO_2_ gas response.

## 2. Materials and Methods

Materials Preparation. GO was prepared via a modified Hummers method [25] starting from graphite flakes of 500 μm maximum size. Monolayers with average sizes of several micrometres and thicknesses of less than 2 nm were obtained and dispersed in water to reach a final concentration of 0.05 mg/mL. Two-dimensional WS_2_ flakes, with average lateral size of 200 nm and average thickness under 15 nm, were obtained by starting from WS_2_ commercial powder (Sigma-Aldrich 243639-50G) exfoliated by ball milling assisted sonication, and dispersed in ethanol and subsequently centrifuged as described in the Supporting Figure S1. Finally, equal amounts of the two GO/water and WS_2_/ethanol solutions were mixed together and sonicated for 10 min to homogenize the dispersion and avoid agglomeration.

Microstructural and chemical characterization. TEM and STEM pictures of the WS_2_-decorated GO were acquired using a TEM—JEOL 2100 Field Emission Transmission Electron Microscope operating at 200 kV by drop casting the dispersion on a lacey grid. Samples prepared by drop casting the WS_2_-GO dispersion on Si_3_N_4_ substrates were further analyzed by X-Ray Photoemission Spectroscopy (XPS) using a PHI 1257 spectrometer equipped with a monochromatic Al Kα source (hν = 1486.6 eV) with a pass energy of 11.75 eV (93.9 eV survey), corresponding to an overall experimental resolution of 0.25 eV. Thin-film XRD measurements were performed at 3° incidence using a Philips PW1710 diffractometer equipped with grazing-incidence X-ray optics, using Cu Kα Ni-filtered radiation at 30 kV and 40 mA.

Sensor fabrication and gas sensing measurements: Thin layers have been prepared by drop casting 8 μL of the water/ethanol dispersed WS_2_-GO solution on Si_3_N_4_ substrates provided with 30 μm spaced Pt interdigitated electrodes, followed by annealing at 70 °C for 30 min to partially reduce the GO to rGO in order to fix the film resistivity in the range of 10^4^–10^5^ Ohm. The electrical resistance of the sensors was measured by an automated system. The sensors placed inside a Teflon chamber (500 cm^3^), provided with Teflon tubings have been exposed to different gas concentrations in the range 200 ppb-10 ppm NO_2_ obtained by mixing certified NO_2_ mixtures with dry air carrier gas at 500 sccm/min flow rate, by means of an MKS147 multi gas mass controller. Electrical resistance was measured by a volt-amperometric technique (AGILENT 34970A) under dark and Purple Blue (PB) light irradiating conditions (PB λ = 430 nm) and different power densities at 270 μW/cm^2^, 468 μW/cm^2^ and 668 μW/cm^2^. The device temperature has been controlled by heating elements and temperature sensors (thermocouples) integrated on the device backside, guaranteeing that the gas pressure was not affected by the changes in the OT.

In this paper the relative response *RR* is represented by the ratio *RR* = *Ra*/*Rg* where *Ra* and *Rg* are the resistances in dry air and in gas respectively and τ_ads_ and τ_des_ (respectively adsorption and desorption times) represent the time required to reach 90% of the full response at equilibrium, during both gas adsorption and desorption.

## 3. Results and Discussion

### 3.1. Morphological and Compositional Characterization of the WS_2_-Decorated rGO

Thin films of WS_2_-decorated rGO deposited on Si_3_N_4_ substrates provided with Pt finger type electrodes have been characterized. We have firstly deposited by drop deposition the minimum amount of WS_2_–rGO solution, corresponding to the formation of a continuous percolation path of GO flakes. The development of a continuous percolation path was assessed by recording the electrical resistance of the film corresponding to the onset of an electrical contact between the electrodes (30 µm apart). By further annealing at 70 °C in air for 30 min, the GO flakes have been partially reduced to rGO to yield baseline resistances in air in the range of 10^4^–10^5^ Ω.

**Figure 1:**
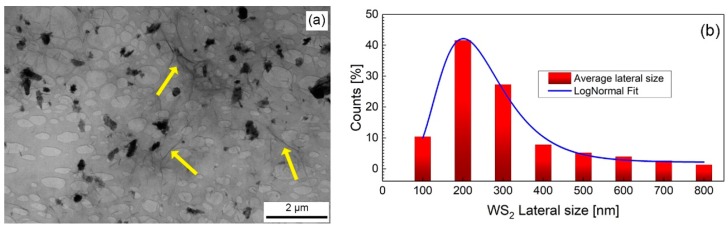
(**a**) Low-resolution TEM image of WS_2_-decorated GO deposited on a lacey grid. WS_2_ flakes (darker regions) distributed over interconnected rGO flakes (light-grey background). The occurrence of grey lines (highlighted by yellow arrows) attest the formation of rGO folded edges; (**b**) Lateral size distribution of WS_2_ flakes and corresponding Log Normal fit.

The WS_2_-decorated rGO morphology was first characterized by low-resolution TEM, as shown in Figure 1a. WS_2_ flakes (darker regions) are distributed over a thin, continuous and uniform layer made of interconnected rGO flakes (light-grey background) as revealed by the presence of grey lines attesting the formation of rGO folded edges (yellow arrows in Figure 1a). Statistical image analysis carried out on differently prepared samples over an area of 80 µm^2^, as shown in Figure 1b, exhibits a log-normal WS_2_ average particle size distribution with an average particle size dimension of 200 nm and an average WS_2_ flakes coverage percentage of 6%. Notably, the dispersion of the WS_2_ flakes is homogeneous over the investigated area, meaning that the sonication step after mixing represents an effective strategy to avoid agglomeration of the WS_2_ flakes.

**Figure 2:**
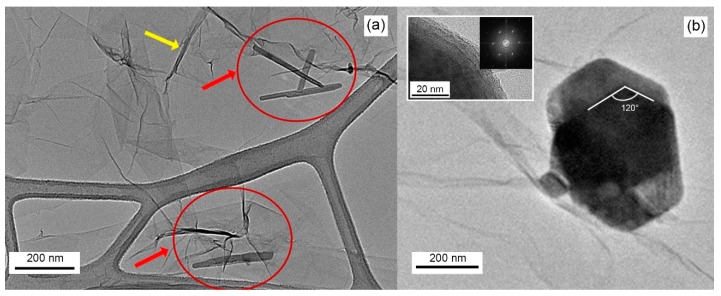
High-resolution TEM of WS_2_-decorated rGO showing: (**a**) light-grey background of interconnected GO flakes. Darker grey lines (yellow arrow) corresponding to folded GO edges and some WS_2_ flakes vertically placed on GO flakes (needless inside the red circle); (**b**) a big hexagonal WS_2_ flake. The inset shows a magnification of the flake’s edges with related SAED analysis pattern.

The TEM analysis shown in Figure 2a reveals the presence of large (i.e., hundreds of nanometers) transparent, irregularly shaped GO flakes with folded edges (yellow arrow) distributing according to a continuous planar underlying layer. From Figure 2a it is shown that WS_2_ flakes may also align vertically, as highlighted by the darker needle-shaped formations inside the red circles of Figure 2a. Statistical analysis carried out on the vertically aligned WS_2_ flakes revealed an average thickness of 15 nm, corresponding approximately to 25 layers. TEM image depicted in Figure 1b illustrates the formation of well-shaped, stacked-few-layers WS_2_, with edge angles of 120°, deposited over the underlying GO flakes. The crystalline nature of few-flakes WS_2_ is confirmed by the selected area electron diffraction (SAED) pattern shown in the inset of Figure 2b, which clearly exhibits the formation of WS_2_ nanosheet with hexagonal atomic arrangements, assigned to WS_2_ (100) plane [26].

**Figure 3:**
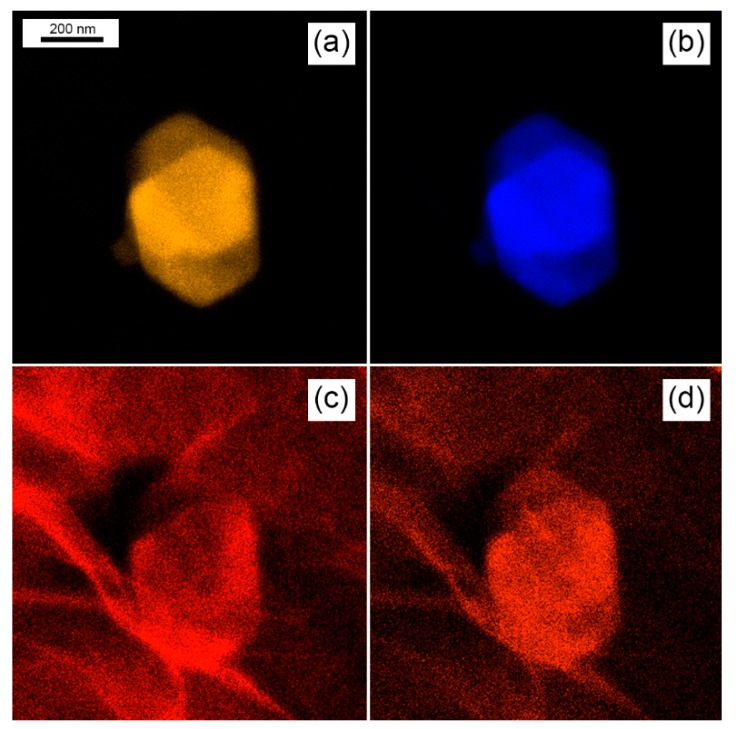
STEM elemental mapping of the flake shown in Figure 2b showing the atomic distribution of: (**a**) sulfur, (**b**) tungsten, (**c**) carbon and (**d**) oxygen.

Figure 3 shows the atomic distribution of sulphur (a), tungsten (b), carbon (c) and oxygen (d) elements, as measured by STEM technique, respectively on the sample displayed in Figure 2b. From Figure 3a,b, it turns out that the distribution of tungsten and sulphur exactly replicates the shape of the WS_2_ flake. The carbon signal over the WS_2_ particles of Figure 3c, may be possibly be attributed to a partial contamination of the sample, whereas the carbon signal deriving from the background, clearly replicates the morphology of the rGO underlying layer. The chromatic signal intensity of oxygen corresponding to the WS_2_ flake (Figure 3d), is slightly brighter than the background, indicating the occurrence of a higher oxygen concentration over the WS_2_ flake with respect to the rGO background, suggesting the occurrence of an oxidation process of WS_2_, as it will be confirmed in the next XPS section.

**Figure 4:**
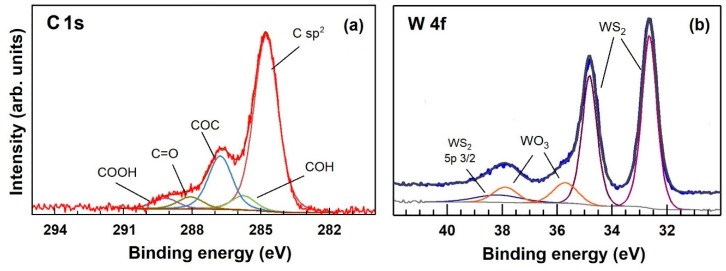
XPS spectra of the WS_2_-decorated rGO showing (**a**) C1s core level spectrum of rGO flakes and (**b**) W 4f core level spectrum of WS_2._

Figure 4 shows the XPS C1s (a) and W4f (b) core level photoemission spectra of WS_2_-decorated rGO air-annealed at 70 °C. According to the literature [27,28], the C1s spectrum shown in Figure 4a has been successfully fitted by the sum of five components assigned to C sp^2^ atoms belonging to aromatic rings and hydrogenated carbon (C=C/C−C 284.7 eV), hydroxyl groups (C−OH, 285.8 eV), epoxy groups (C−O−C, 286.8 eV), carbonyl groups (C=O, 288.0 eV), and carboxyl groups (C=O(OH), 289.2 eV). Upon thermal annealing at 70 °C, the intensity of all the oxygen-containing groups is lowered, with regard to sp^2^ carbon containing ones (i.e., (C=C/C−C), signifying a loss of oxygen in favor of sp^2^ carbon. Compared to previous results [29], the XPS spectrum shown in Figure 4a is located halfway between the XPS signals of as deposited GO and the one corresponding to 200 °C UHV-annealed rGO. Regarding the tungsten W4f of Figure 4b, the four peaks fitting the spectrum can be located, according to the literature [30,31], to WS_2_ and WO_3_. These results suggest, as previously reported for MoS_2_ and WS_2_ [5,7,11], that crystalline WS_2_ is partially oxidized to WO_3_ with an associated content of approximately 18%. Gracing incidence XRD measurements on the WS_2_-decorated rGO annealed at 70 °C for 30 min, as shown in the supporting Figure S2, revealed that the WO_3_ is amorphous.

### 3.2. NO_2_ Gas Response

**Figure 5:**
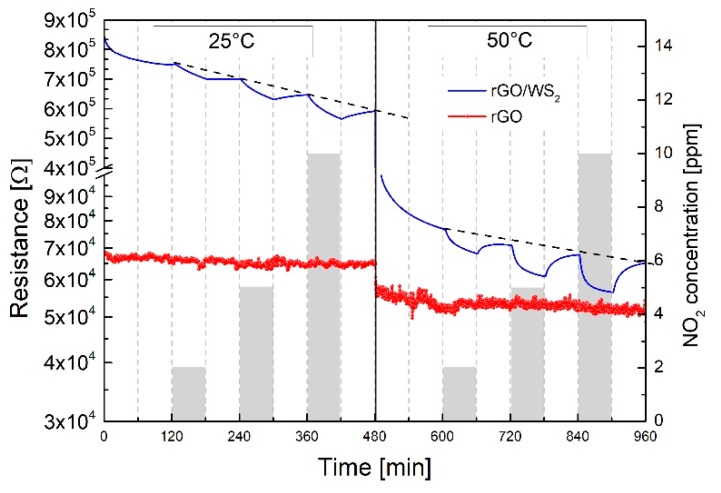
Electrical responses of single rGO (red line) and WS_2_-decorated rGO films (blue line) in dry air and NO_2_ concentrations in the range 2–10 ppm at 25 °C and 50 °C operating temperature.

The reacting surface of the WS_2_-decorated rGO comprises, as attested by microstructural characterization, a flat underlying layer made of large and interconnected rGO flakes covered with dispersed, partially oxidized, WS_2_ flakes. Given that WS_2_ flakes do not form a continuous layer (see Figure 1a), it is the rGO layer which mostly determines the baseline resistance of the WS_2_–rGO hybrid. The NO_2_ gas responses in dry air of a single rGO film and a WS_2_-decorated rGO film at 25 °C and 50 °C operating temperatures (OT) are compared in Figure 5.

By increasing the OT to 50 °C, the baseline resistance (BLR) of the WS_2_-decorated rGO (dotted lines in the figure) decreases almost of one decade as compared to rGO, attesting the substantial contribution of the WS_2_ semiconductor to the overall resistance response. At 25 °C OT, by increasing the NO_2_ gas concentration, it is noted that: (i) the baseline resistance (BLR) is not recovered after gas desorption; (ii) baseline resistance steadily drifts, decreasing its resistance; (iii) the electrical signal never reaches equilibrium under adsorption/desorption conditions within the time schedule of the experiment (i.e., 60 min). Conversely, by increasing the OT to 50 °C, WS_2_-decorated rGO shows a faster response, improved equilibrium conditions and reduced baseline drift. Besides the positive effects related to the OT, it may be concluded that irreversible adsorption and baseline drift phenomena are still evident at 50 °C OT, representing a serious drawback for the exploitation of near room temperature WS_2_–rGO hybrids sensors.

**Figure 6:**
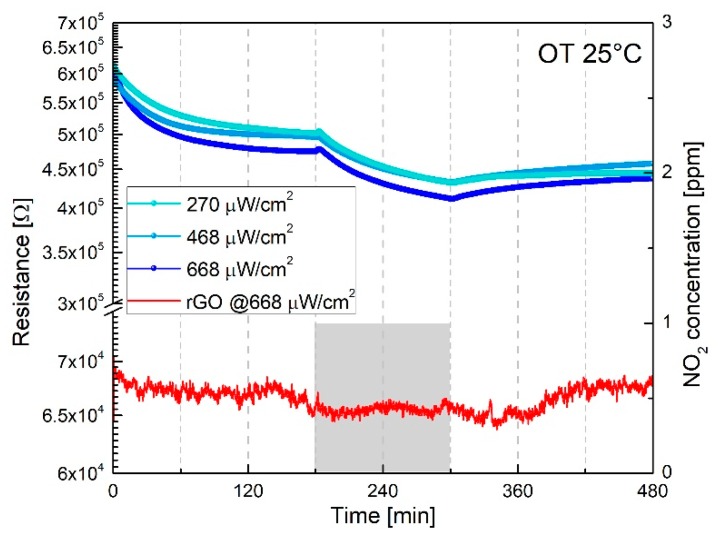
Response to 1 ppm NO_2_ in dry air of WS_2_-decorated rGO and bare rGO film (red curve) irradiated by Purple–Blue (λ = 430 nm) light at different power densities (270 μW/cm^2^, 468 μW/cm^2^, 668 μW/cm^2^).

According to previous research which demonstrated the positive effect of the combined action of light irradiation and thermal activation on the NO_2_ response of WO_3_ and NiO semiconductor sensors [12,13], Figure 6 shows the rGO’s and WS_2_–rGO hybrid’s NO_2_ gas responses at 25 °C in dry air under Purple Blue light (PB λ = 430 nm @ 2.88 eV) and different power densities at 270 μW/cm^2^, 468 μW/cm^2^ and 668 μW/cm^2^. Bare rGO exhibits neither significant changes of the electrical response at different power densities (in Figure 6 the response at 668 μW/cm^2^ is shown) nor any appreciable gas response to 1 ppm NO_2_. WS_2_-decorated rGO yields relative responses (*RR* = *Ra*/*Rg* at 1ppm NO_2_) of approximately 1.21, more or less independent of the power density. Response and recovery times, in contrast, improve with increasing the power density.

**Figure 7:**
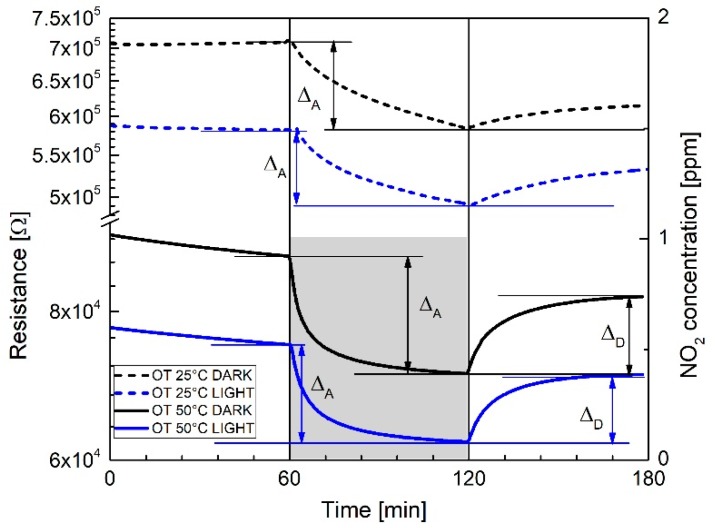
The 1 ppm NO_2_ gas responses of the WS_2_-decorated rGO under “dark” and “light” conditions (dotted lines) at 25 °C operating temperatures compared to the “dark” and light” conditions (solid lines) at 50 °C operating temperature.

By selecting the 668 μW/cm^2^ light power density, Figure 7 shows the electrical response of the WS_2_-decorated rGO to 1 ppm NO_2_ in dry air under “dark” and “purple–blue” conditions at 25 °C and 50 °C OT respectively.

If we now define the recovery percentage (*RP*) as the percentage ratio (Δ*_D_*/Δ*_A_*) × 100, where Δ*_D_* and Δ*_A_* (see Figure 7) are the desorption/adsorption resistances’ variations (Δ), measured at the end of each desorption/adsorption cycle (i.e., 60 min), it turns out that light illumination strongly enhances the recovery of the baseline resistance during desorption.

As shown in Figure 7 and Table 1, recovery percentage (RP) values increase from 23% (dark) to 65% (light) at 25 °C OT, and from 60% (dark) to 70% (light) at 50 °C OT. Moreover, considering features and shapes of the curves displayed in Figure 7, at 25 °C OT regardless of “dark” or “light” exposures, no equilibrium conditions are achieved within the timescale of the experiment (i.e., 60 min). In contrast, as attested by the horizontal slopes of the two bottom curves of Figure 7, equilibrium conditions are achieved under adsorption/desorption conditions when light irradiation is performed and when the OT is increased to 50 °C. Notably, according to Table 1, given the associated uncertainty of the measurement (± 0.02), no substantial changes of the relative responses’ values are recorded with respect to 25 °C, either by increasing the OT to 50 °C or by illuminating the sensor with purple blue light.

The results shown in Table 1 are in line, both in terms of sensitivity, using comparable definitions, and time constants with those reported in literature for hybrid graphene/MoS_2_ structures for NO_2_ sensing [18,19] working in dark conditions and at higher operating temperatures (at least 150 °C).

**Figure 8:**
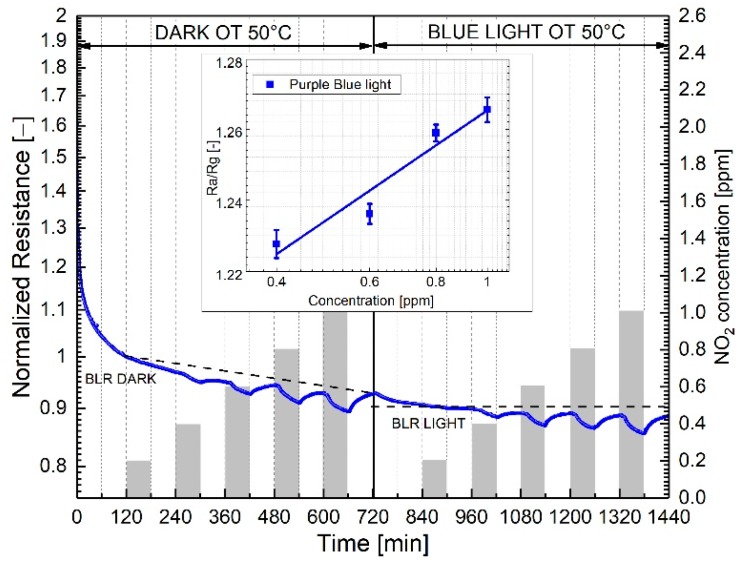
Comparison of WS_2_-decorated rGO’s electrical responses to increasing NO_2_ concentrations at 50 °C operating temperature under dark and light conditions respectively. The inset shows the sensitivity plot corresponding to light conditions. The bars in the inset represent the standard deviation calculated over a set of five measurements performed for each gas concentration. Dotted lines indicate the baseline resistance in dry air.

Figure 8 compares the normalized dynamic gas responses of the WS_2_–rGO sensor at 50 °C OT under dark and light conditions, respectively. By increasing the NO_2_ concentration from 200 ppb to 1 ppm, purple blue light irradiation promotes full baseline recovery after each NO_2_ pulse, as demonstrated by the horizontal slope of the dotted line of Figure 8. WS_2_-decorated rGO exhibits an experimental detection limit of 400 ppb NO_2_ in dry air. The inset of Figure 8 shows the sensitivity plot of the response under PB illumination, with associated standard deviations. Reproducibility tests of the electrical response under pulse and cumulative NO_2_ adsorption/desorption exposures, shown in the supporting Figure S3, exhibit no substantial irreversible adsorption phenomena, as well as a fairly good reproducibility of the electrical response. Additionally, long-term stability properties of the baseline and saturation resistances to 1 ppm NO_2_ over a period of 12 months were also recorded. Supporting Figure S4 shows baseline resistances (upper curve) and saturation resistances corresponding to 1 ppm (lower curve), randomly collected over a period of 52 weeks, with associated standard deviations calculated over a set of five consecutive measurements. No remarkable fluctuations of both baseline and resistances at saturation are detectable, demonstrating good long-term stability properties of the WS_2_ films.

**Figure 9:**
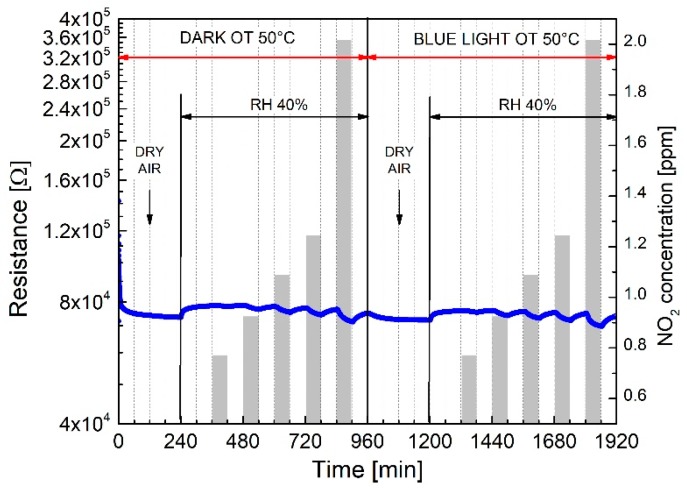
The influence of 40% Relative Humidity (40% RH) on the NO_2_ response of WS_2_-decorated rGO under dark and light illumination at 50 °C operating temperature.

The effect of 40% relative humidity (RH) to the NO_2_ response, under dark and light conditions, at 50 °C OT, is shown in Figure 9. Regardless of the illumination: (i) the baseline resistance slightly increases from dry to humid (40% RH) conditions; (ii) the NO_2_ relative responses (*Ra*/*Rg*) at 40% RH, do not change appreciably neither under dark or light conditions; (iii) the baseline resistance is fully recovered after each adsorption/desorption cycle. This behavior demonstrates that humidity, regardless of the illumination conditions, does not appreciably interfere with the NO_2_ gas adsorption mechanism, suggesting, as it will be discussed in the next paragraph, that NO_2_ preferentially adsorbs on WS_2_, since energetically favored as respect to water vapor.

Finally, the sensors’ selectivity among other interfering gases such as H_2_, NH_3_, acetone and ethanol has been recorded and the results shown in Supporting Figure S5. The WS_2_-decorated rGO sensor exhibits an excellent selectivity for NO_2_ gas as compared to the other investigated species, making it suitable for being a selective NO_2_ sensor.

#### Gas Sensing Mechanism

Discussing the gas sensing mechanism of the WS_2_–rGO hybrid sensor with respect to water vapor and NO_2_, the single contribution of the rGO and WS_2_ species and WS_2_–rGO hybrid to the overall gas response have to be considered.

Regarding the single rGO in our previous work [29] we demonstrated that both as-deposited GO and partially reduced rGO (annealed in vacuum at 200 °C) exhibit a *p*-type response to NO_2_, resulting in a decrease of the resistance in the operating temperature range of 25–150 °C. Considering that the rGO prepared in this work shows a degree of reduction, located halfway between these two extremes, we would have expected a decrease of the resistance in Figure 5, rather than no response when exposing bare rGO film to NO_2_ gas. The lack of any gas response may not be entirely attributed to the decrease of functional groups induced by the mild reduction process (i.e., air annealed at 70 °C), but mostly to a substantially smaller amount of the deposited rGO, which amounts to approximately 1/10 of the quantity previously utilized [29]. It may be concluded that the deposition procedure adopted here yields an rGO film which does not significantly contribute to the NO_2_ response, while it guarantees the formation of a continuous, conductive layer “bridging” distant platinum finger-type electrodes.

Regarding single WS_2_, which is reported to increase its resistance to NO_2_, exhibiting an *n*-type response [7,16], some considerations apply. According to the XPS section, WS_2_ flakes deposited on rGO partially oxide to amorphous WO_3_ (approx 18%). Amorphous WO_3_, as previously discussed [7,16], acts as a non-conductive phase, eventually inhibiting the charge–carrier transfer mechanism within the flakes. It turns out that the formation of WO_3_, while not contributing to the overall gas response, has the merely negative effect of partially covering the underlying reacting surface of the WS_2_ flakes, eventually decreasing the relative gas response.

Regarding crystalline WS_2_, the variation of the electrical resistance induced by Air–NO_2_ mixtures, is part of a complex mechanism involving the combined effects physisorption, chemisorption, the role of edges and surface defects and the transduction mechanism. First principle calculations on MoS_2_ sulphur-vacancy-defective monolayers [32,33] demonstrated that O_2_ firstly chemisorbs on existing sulphur vacancies, secondly, that sulphur vacancies are passivated (i.e., “healed”) by the dissociative chemisorption of O_2_ molecules, leading to the formation of two Mo-O bonds. In case of direct NO_2_ molecules interaction with sulphur vacancies, a dissociative chemisorption of NO_2_ takes place, leading to oxygen atoms passivating the vacancies (i.e., “secondary healing”) and NO molecules eventually to be physisorbed on the MoS_2_ surface. Considering that both MoS_2_ and WS_2_, are susceptible to spontaneous oxidation in air [11], the mechanism of sulphur vacancies suppression operated by O_2_ and NO_2_ claimed for MoS_2_, can be reasonably extended to sulphur defective WS_2_. Under these circumstances, literature reports based on first principle calculations on “healed” WS_2_ surfaces pointed out that O_2_, NO_2_ and H_2_O physisorb on defect-free monolayer WS_2_ surfaces [34,35].

Regarding WS_2_ –rGO hybrids, they decrease their overall resistance when exposed to NO_2_ (see Figure 5), exhibiting, as reported for MoS_2_/Graphene composites [18,20], an overall *p*-type response. Bearing in mind that the WS_2_–rGO hybrid’s gas response depends on both charge transfer values (positive charge transfer values between adsorbing molecules and the material surface stand for electrons withdrawal from the material) and adsorption energies (negative adsorption energy means that the adsorption process is exothermic and energetically favourable), the following discussion applies.

According to Table 2, adsorption of a single NO_2_ molecule on rGO yields charge transfer values of approximately 0.029e and associated adsorption energies of −0.80 eV [32,34,35]. By contrast, the adsorption of a single NO_2_ molecule on defect free WS_2_ indicate that NO_2_ physisorbs on WS_2_ yielding charge transfer values of 0.178e and associated adsorption energies of −0.41 eV [34,35].

Considering that calculated NO_2_ charge transfer values are 0.029e for rGO and 0.178e for WS_2_, it turns out that WS_2_ yields a net charge transfer 6.14 times higher than on of rGO per NO_2_ physisorbed molecule. Given these premises, the electrical response of WS_2_-decorated rGO as compared to single rGO shown in Figure 5, accounts for the larger WS_2_′s charge carrier exchange with respect to rGO.

**Figure 10:**
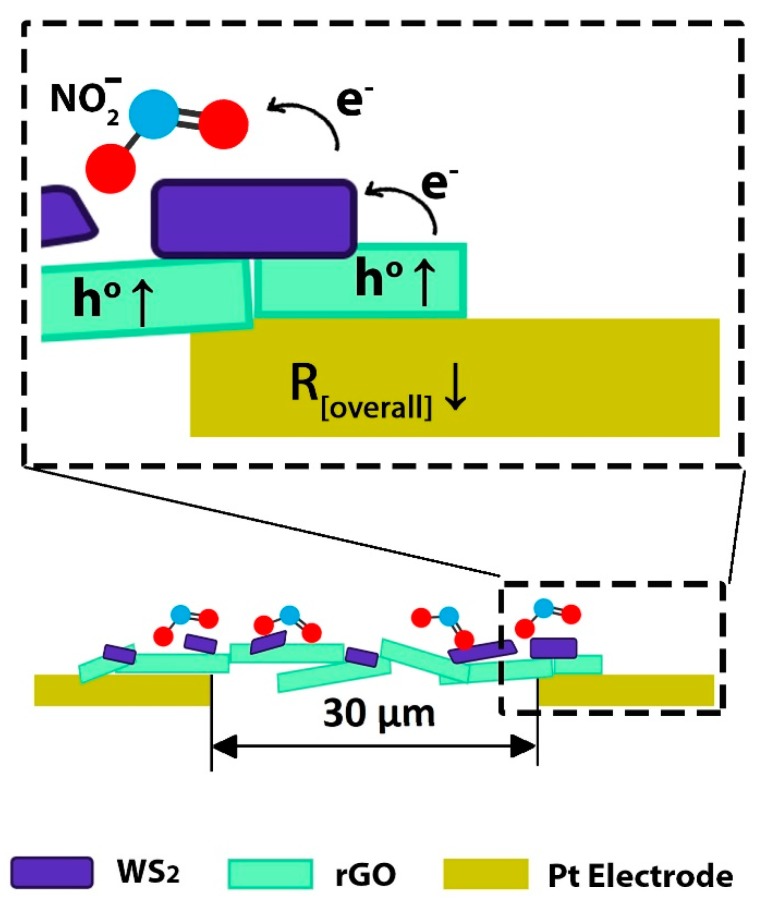
Schematic illustration of the proposed sensing mechanism of WS_2_-decorated rGO hybrid during NO_2_ exposure.

Figure 10, which is a schematic illustration of the WS_2_-decorated rGO film, deposited between two platinum electrodes 30 µm apart, highlights that NO_2_ molecules adsorbs on both rGO and WS_2_ causing electrons to withdraw from both materials. Considering the smaller charge transfer induced by NO_2_ adsorption on rGO (i.e., 0.029e) with respect to WS_2_ (i.e., 0.178e), by exposing the WS_2_–rGO hybrid to NO_2_ gas, the NO_2_ gas molecule withdraws electrons mainly from the WS_2_ surface. As a consequence, electron-depleted *n-type* WS_2_ flakes drain electrons from the underlying *p-type* rGO, thanks to a rapid electron transport from the highly conducting rGO to the less-conducting WS_2_ [19,22]. The increase of hole concentration in *p-type* rGO due to physisorption of NO_2_ molecules on WS_2_ flakes, explains the decrease of the overall resistance of the *p*-*type* WS_2_-decorated rGO shown in Figure 5. It may be concluded that rGO flakes, with excellent transport capability, serve as highly conductive channels bridging distant electrodes, whereas WS_2_ decoration eventually modulates the NO_2_ gas response.

Regarding the influence of purple–blue light, it is well known that response and recovery rates depend on the adsorption energy of the adsorbed gas molecules (see Table 2). Light irradiation and thermal activation represent, therefore, two alternative or complementary modes to improve response/recovery rates [13]. By irradiating WS_2_-decorated rGO with purple–blue light (PB λ = 430 nm) the associated photon energy of 2.88 eV and power density of 668 μW/cm^2^ provide: (i) a large quantity of photo generated electrons/holes; (ii) the required energy to desorb physisorbed O_2_, NO_2_ and H_2_O molecules from both rGO and WS_2_ flakes. Under NO_2_ gas and PB light illumination, physisorbed oxygen partially desorbs from its adsorption site (☐) according to Reaction (1) while NO_2_ physisorbs on free sites (☐) left behind from oxygen desorption according to Reaction (2). At equilibrium the overall reaction is represented by Equation (3).

(1) $$\left(O_{2}^{-}-☐\right)+h^{+}+e^{-}⇌↑O_{2}+☐+e^{-}$$

(2) $$NO_{2}+☐+e^{-}⇌\left(NO_{2}^{-}-☐\right)$$

(3) $$\left(O_{2}^{-}-☐\right)+h^{+}+e^{-}⇌\left(NO_{2}^{-}-☐\right)+↑O_{2}$$

Considering that NO_2_ molecules withdraw electrons with an associated charge transfer of 0.178e as compared to that of Oxygen of 0.136e [34], under NO_2_ adsorption, Equation (3) is shifted to the right, meaning an excess of holes, which explains the overall resistance decrease of the WS_2_–rGO hybrid. Moreover, the improved recovery percentages and response times of Figure 7, account for the extra light-photogenerated carriers which speed up the time to reach equilibrium (Equation (3)) both during gas exposure and recovery.

Regarding water interaction with WS_2_–rGO hybrid the reason why humidity, regardless of the illumination conditions, does not appreciably interfere with the NO_2_ gas response, can be tentatively explained (see Table 2) evaluating that the adsorption energy of a NO_2_ molecule on WS_2_ is approximately twice (−0.41 eV) the corresponding energy of water (−0.23 eV), indicating a stronger attitude of NO_2_ molecules to adsorb on WS_2_ compared to water. Finally, the initial resistance increase of the WS_2_–rGO when exposed to humid air can be explained taking into consideration that water is a reducing agent, which adsorbs on both rGO and WS_2_ injecting electrons, thus decreasing the hole concentration in rGO and WS_2_, causing an overall resistance increase of the WS_2_–rGO hybrid.

## 4. Conclusions

We have exfoliated, by a combined grinding and sonication technique, WS_2_ commercial powders into mono-to few-layer flakes of WS_2_, with an average dimension of 200 nm, which have been successfully dispersed with rGO flakes with average dimensions of 700 nm, to yield WS_2_-decorated rGO as chemo-resistive NO_2_ thin film sensor. Operating at near room temperature conditions and providing an extra source of energy, by purple–blue light illumination, we have proposed a possible strategy to improve adsorption/desorption rates and to suppress water vapour cross sensitivity. The deposition procedure adopted here yields WS_2_ flakes which mostly drive the NO_2_ gas response, while the underlying rGO film guarantees the formation of a continuous, conductive layer “bridging” distant platinum finger-type electrodes. By retrieving literature data about charge carriers and adsorption energies deriving from the interaction of NO_2_, O_2_ and water molecules with WS_2_ and rGO, we have proposed a gas sensing mechanism which accounts for the overall gas response of the WS_2_–rGO hybrids.

## Figures and Tables

**Table 1:** Comparison of *RR*, *RP*, τads and τdes to to 1 ppm NO_2_ in dark conditions and PB light illumination (λ = 430 nm at 668 µW/cm^2^) at different OT (25–50 °C). Notably (-) means that no equilibrium conditions have been reached within the time scale of the experiment.

Response TO 1 ppm NO_2_				
Operating Conditions	*RR* *Ra*/*Rg*	*RP* Δ*_D_*/Δ*_A_*	τ _ads_	τ _des_
	(–)	(%)	(min)	(min)
25 °C DARK	1.18 ± 0.02	23	-	-
25 °C LIGHT	1.21 ± 0.02	65	-	-
50 °C DARK	1.20 ± 0.02	60	22	26
50 °C LIGHT	1.27 ± 0.02	70	16	18

**Table 2:** Adsorption energy on rGO and WS_2_ of O_2_, NO_2_ and H_2_O molecules. Note that negative adsorption energy means that the adsorption process is exothermic and energetically favorable.

Physisorbed Molecules	Adsorption Energy on rGO (eV)	Reference	Adsorption Energy on WS2 (eV)	Reference
O2	−0.16	[36]	−0.24	[36]
NO2	−0.80	[32,34,35]	−0.41	[34,35]
H2O	−0.04	[37]	−0.23	[34]

## Supplementary Materials

The following are available online at https://www.mdpi.com/1424-8220/19/11/2617/s1, Figure S1: Schematic illustration of WS2 exfoliation process, Figure S2: Grazing incidence XRD spectrum of WS_2_-decorated rGO film, Figure S3*:* Reproducibility and base line recovery features of the WS_2_-decorated rGO by exposing the film to both dynamic and cumulative NO_2_ concentrations (5–10 ppm) under light irradiation (purple blue), Figure S4. Long term stability properties of the WS_2_/rGO hybrid sensor, Selectivity response of the the WS_2_/rGO hybrid sensor to different oxidizing and reducing gases, measured at 50 °C operating temperature under purple-blue illumination.
