# Supplementary material for: Near Room Temperature Light-Activated WS2-Decorated rGO as NO2 Gas Sensor
Source: Sensors (Basel). 2019 Jun 9;19(11):2617. doi: 10.3390/s19112617 (PMC6603724; doi:10.3390/s19112617)
Supplement: Supplementary file 1 [file sensors-19-02617-s001.pdf]

SUPPLEMENTARY MATERIAL

Near Room Temperature Light Activated WS_2-decoreated rGO as NO_2 Gas Sensor

Valentina Paolucci [1,*], Seyed Mahmoud Emamjomeh [1], Luca Ottaviano [2,3] and Carlo Cantalini [1]

[1] Department of Industrial and Information Engineering and Economics, Via G. Gronchi 18, University of L'Aquila, I-67100 L'Aquila, Italy; seyedmahmoud.emamjomeh@graduate.univaq.it (S.M.E.), carlo.cantalini@univaq.it (C.C.)
[2] Department of Physical and Chemical Sciences, Via Vetoio 10, University of L'Aquila, I-67100 L'Aquila, Italy; luca.ottaviano@aquila.infn.it (L.O.)
[3] CNR-SPIN Uos L'Aquila, Via Vetoio 10 I-67100 L'Aquila, Italy
* Correspondence: valentina.paolucci2@graduate.univaq.it; Tel.: +39-328-952-5503

Supplementary Figures

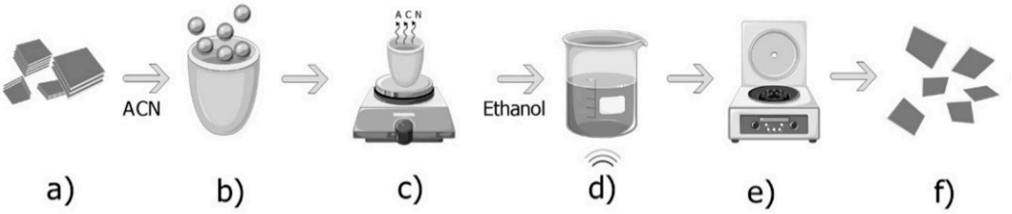

Figure S1. Schematic illustration of WS_2 exfoliation process. WS_2 commercial powder was dispersed in Acetonitrile and ball milled (b) in a planetary milling machine. The ACN residuals were then evaporated overnight at room temperature (c). After ACN evaporation, the ball milled powder was dispersed in pure ethanol and probe sonicated (d) in a cool bath (T 25°C). Finally, the sonicated dispersion was centrifuged (e) and the supernatant collected (f). Some drawings of this figure are distributed by "Servier Medical Art by Servier".

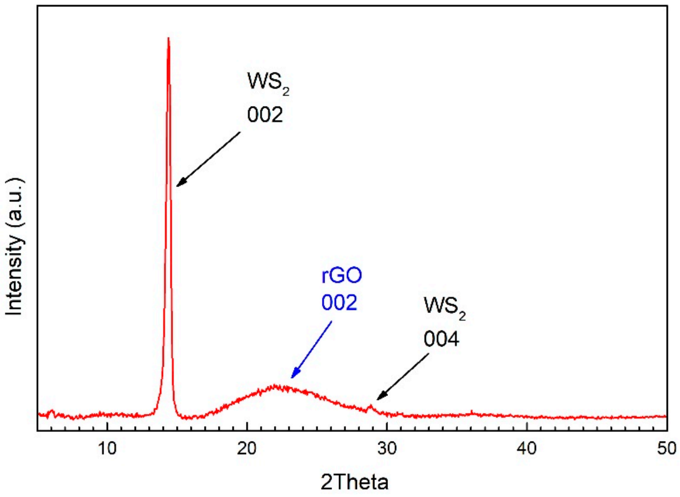

Figure S2. Grazing incidence XRD spectrum of WS_2-decorated rGO film.

According to literature [1,2] the typical WS_2 peaks at 2Theta 14.3 and 28.8 representing the (002) and (004) planes and the broad peak in the region 2Theta 20-25 attributed to rGO's (002) are displayed. No evidence of crystalline WO_3 is detected.

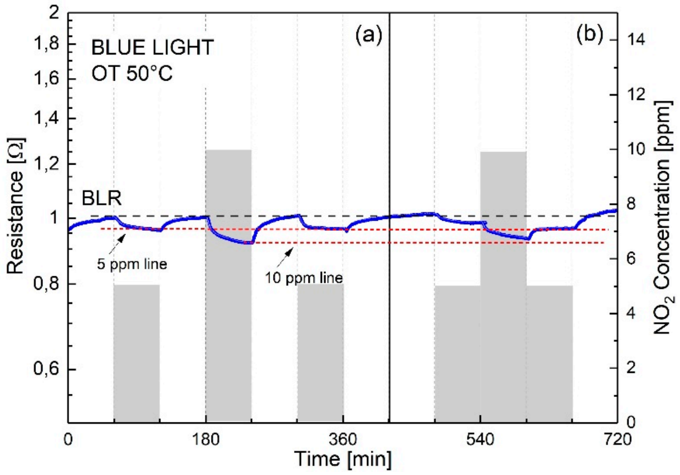

Figure S3. Reproducibility and base line recovery features of the WS_2-decorated rGO by exposing the film to both dynamic and cumulative NO_2 concentrations (5–10 ppm) under light irradiation (purple blue). Panel (a): dynamic responses to repeated on/off runs; panel (b): cumulative adsorption/desorption runs by stepwise increase and decrease of the NO_2 concentration.

The WS_2-decorated rGO film shows good dynamic and cumulative responses to NO_2. Supporting Figure S3 (a) displays the electrical signal under forward and backward dynamic pulses to NO_2 in the concentration range 5–10 ppm. BLR fully regains its initial value after completion of each cycle. Under cumulative stepwise adsorption/desorption mode, as shown in Supporting Figure S3 (b), the NO_2 gas resistance increase/decreases steadily matching almost the same resistances at saturation and in comparison, to panel (a).

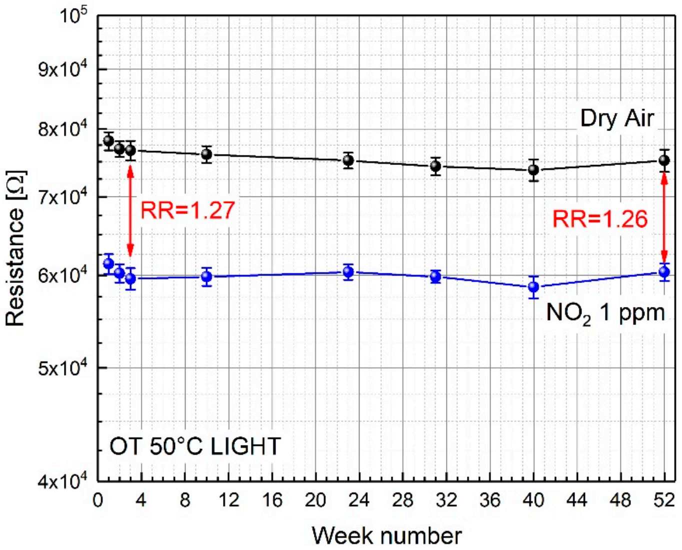

Figure S4. Long term stability properties of the WS_2/rGO hybrid sensor. Base line resistances (upper curve) and saturation resistances corresponding to 1 ppm (lower curve), randomly collected over a period of 52 weeks, with associated standard deviations calculated over a set of 5 consecutive measurements are shown. Relative responses (RR) taken over the investigated period are also highlighted.

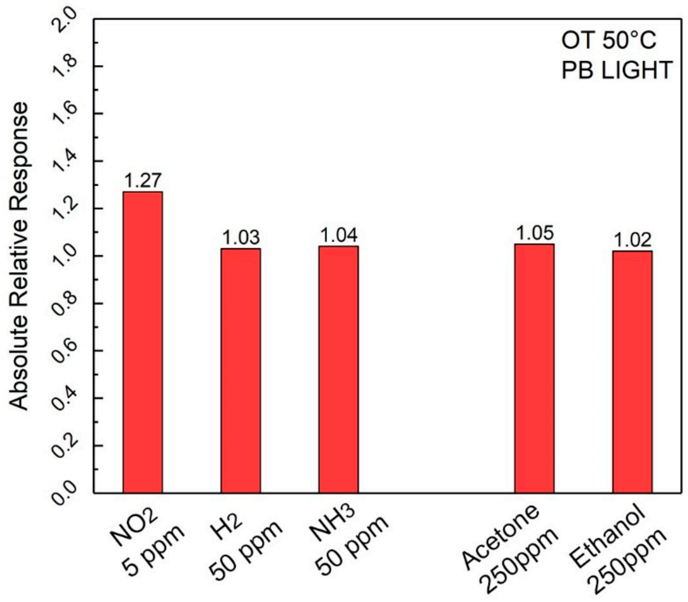

Figure S5. Selectivity response of the the WS_2/rGO hybrid sensor to different oxidizing and reducing gases, measured at 50°C operating temperature under purple-blue illumination.

References

1. Thi Vu, T.H.; Thi Tran, T.T.; Thi Le, H.N.; Thi Nguyen, P.H.; Bui, N.Q.; Essayem, N. A new green approach for the reduction of graphene oxide nanosheets using caffeine. *Bull. Mater. Sci.* **2015**, *38*, 667–671.
2. Zhang, K.; Zhang, Y.; Wang, S. Enhancing thermoelectric properties of organic composites through hierarchical nanostructures. *Sci. Rep.* **2013**, *3*, 3448.
